# Brown is the new green: Discovery of an algal enzyme for the final step of fucoxanthin biosynthesis

**DOI:** 10.1093/plcell/koad138

**Published:** 2023-05-17

**Authors:** Ching Chan

**Affiliations:** Assistant Features Editor, The Plant Cell, American Society of Plant Biologists, Rockville, MD, USA; Department of Life Science, National Taiwan Normal University, Taipei 11677, Taiwan

Carotenoids are essential cofactors for photosynthesis, and some carotenoids, such as peridinin and fucoxanthin found in marine algae, provide a selective advantage by allowing these organisms to capture blue-green light, the main wavelengths that reach deep-sea environments (Sandmann 2021). Ecologically, fucoxanthin-containing brown algae, diatoms, haptophytes, and dinoflagellates are the most abundant algae in the ocean. The reported antidiabetic, antiobesity, anticancer, and antioxidant properties of fucoxanthin have led to steep increases in the cost of purified fucoxanthin (Khaw et al. 2022) and interest in elucidating the enzymes for fucoxanthin biosynthesis. Previously, using a reverse genetics approach in the model diatom *Phaeodactylum tricornutum*, Bai et al. (2022) characterized 2 enzymes, violaxanthin de-epoxidase-like 2 (VDL2) and zeaxanthin epoxidase 1, involved in the synthesis of fucoxanthin, leaving the final biosynthetic steps unidentified. A continuing effort by **Tianjun Cao, Yu Bai, and colleagues (Cao et al. 2023)** has elucidated the final missing step of the pathway, catalyzed by carotenoid isomerase-like protein 5 (CRTISO5).

**Figure 1.**
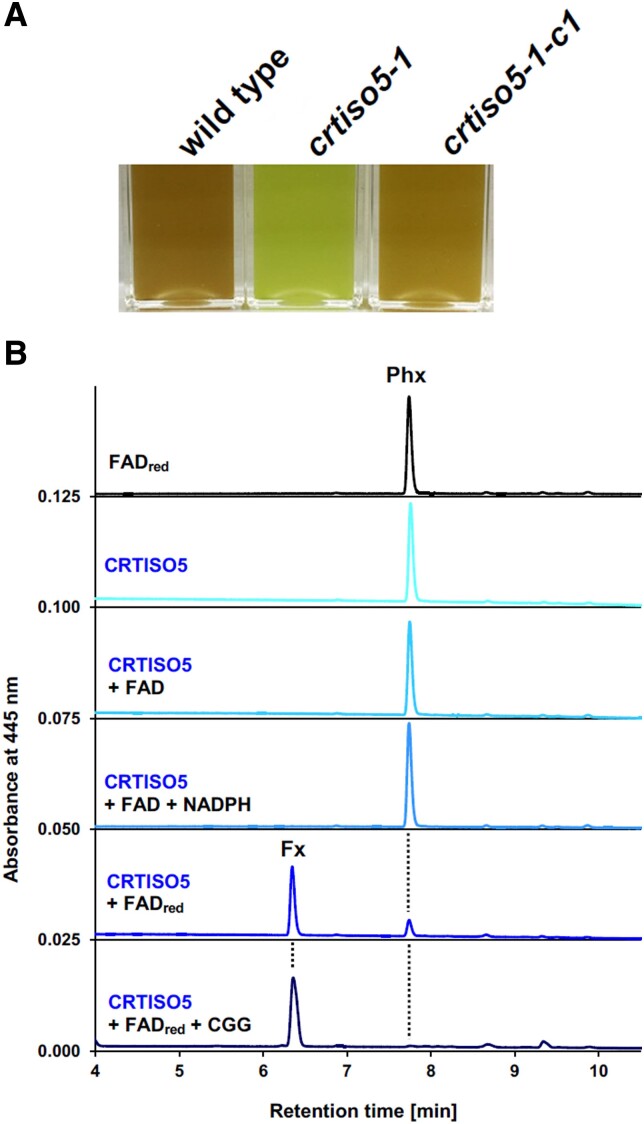
CRTISO5 catalyzes the final step of fucoxanthin biosynthesis in *P. tricornutum*. **A**) Mutation of *CRTISO5* turned the brown algae green. **B**) Addition of recombinant CRTISO5 to phaneroxanthin yielded fucoxanthin without additional carotenoid intermediates. Phx: phaneroxanthin; Fx: fucoxanthin. Adapted from Cao *et al*. (2023) Figure 2A and 3B.

Based on coexpression with VDL2, CRTISO5 was a good candidate to be involved in fucoxanthin synthesis and was targeted for genetic, enzymatic, and structural analyses. Mutation of *CRTISO5* in *P. tricornutum* turned the brown algae green, a typical phenotype for the loss of fucoxanthin production (Fig. 1A) and overaccumulation of its substrate, phaneroxanthin. The mutant was defective in photosynthetic complex assembly, and the efficiency of photosynthesis was reduced. CRTISO proteins are isomerases that catalyze C=C double bond isomerization in cyanobacteria (Breitenbach et al. 2001; Masamoto et al. 2001), Arabidopsis (Park et al. 2002), and tomato (Isaacson et al. 2002). The conversion of phaneroxanthin to fucoxanthin had been assumed to require 2 steps: saturation to an intermediate (dinoxanthin) followed by hydroxylation and spontaneous tautomerization to fucoxanthin. Surprisingly, the addition of recombinant CRTISO5 to phaneroxanthin yielded fucoxanthin directly without any carotenoid intermediates (see Fig. 1). In vitro reaction using heavy water (H_2_^18^O) yielded fucoxanthin with 2 additional atomic mass units, indicating that the conversion of phaneroxanthin to fuxoxanthin involved hydration by water molecules. In silico prediction of the enzyme-substrate interface identified potential active-site residues for site-directed mutagenesis analyses. Out of 5 candidate sites, F291A or Y306A mutations completely abolished CRTISO5 activity, whereas other mutations played relatively minor roles. Overall, CRTISO5 turns out to be the long-sought fucoxanthin synthase, unexpectedly hydrating phaneroxanthin to fucoxanthin.

This finding raises interesting questions regarding the evolution and diversity of the CRTISO family. By phylogenetic analyses complemented with experiments using orthologs from closely related haptophytes, Cao et al. inferred that CRTISO5 hydratase activity evolved from gene duplication and neofunctionalization, which distinguished its function from the common ancestor with CRTISO isomerase activity. This study demonstrates the critical function of CRTISO5 in fucoxanthin biosynthesis, highlighting the importance of fucoxanthin in photosynthesis and opening up new opportunities for the biocatalytic production of fucoxanthin for the healthcare industry.
